# Power of Memory: A Natural Killer Cell Perspective

**DOI:** 10.3390/cells14110846

**Published:** 2025-06-05

**Authors:** Oishi Sinha, SK Abhipsha, Sumit Sen Santara

**Affiliations:** Department of Biological Sciences, Indian Institute of Science Education and Research Kolkata, Mohanpur 741246, India; os22rs064@iiserkol.ac.in (O.S.);

**Keywords:** natural killer (NK) cells, NK memory, cytomegalovirus (CMV), cytokine-induced memory-like (CIML), tumor-induced memory-like (TIML), cancer, leukemia, NK cell-based therapies

## Abstract

Memory is an incredible aspect of our immune system. Similarly to our cognitive memory, it allows us to remember and respond more efficiently to subsequent encounters with the same pathogens, making it possible to act on the information built by previous experiences. This process is critical for the body’s defenses against infections and is the cornerstone for the effectiveness of vaccines. Immunological memory, traditionally considered an exclusive quality of the adaptive immune system, is a sophisticated component of the immune response system that is characterized by the ability to recognize and remember specific pathogens. This form of memory is primarily observed in antigen-specific T and B cells, which are specialized for recognizing particular antigens and generating a quicker immune response upon each successive reinfection over a long period of time. Natural killer (NK) cells, essential as the body’s first line of defense against a wide range of viral infections and tumors, have traditionally been classified as a key component of the innate immune system, characterized by their lack of antigen specificity and memory. However, the concept of innate vs. adaptive has been evolving, with increasing evidence suggesting that specific cellular subsets of the innate immune system may also play a role in immunological memory. This review aims to provide a comprehensive overview of the recent advances in the understandings of the molecular mechanisms driving the development of memory-like properties in NK cells, with a primary focus on human data in the context of various diseases and infectious conditions. Additionally, we will examine the therapeutic implications of these findings, highlighting how insights into NK cell memory can contribute to the development of novel immunotherapies and improve strategies for treating infections, cancer, and autoimmune disorders.

## 1. Introduction

Memory is a term that generally refers to recall and response potential to a multitude of complex stimuli. In the context of immunology, memory is defined as an antibody-specific enhanced response to a secondary exposure that persists long-term in the system. The vertebrate immune system is divided into innate and adaptive arms, and, classically, memory is attributed only to the adaptive immune cells, like B and T cells. NK cells are a subset of lymphocytes classified within the family of innate lymphoid cells (ILCs) and were discovered as a cytotoxic effector in eliminating tumors without any priming, independently by Dr. Kiessling and by Herberman and colleagues. NK cells are known to kill malignantly transformed and virally infected cells mainly through the release of cytotoxic granules containing perforin and granzymes [1]. NK cells also function as potent immunomodulators by secreting a range of immunologically active molecules, including their signature cytokine interferon-gamma (IFN-γ), along with tumor necrosis factor (TNF), granulocyte–macrophage colony-stimulating factor (GM-CSF), and chemokines such as CCL3 and CCL4 [2]. The function of NK cells is determined by the overall input from the germline-encoded activating and inhibitory receptors [3]. Activating receptors, such as NKG2D and NKp46, recognize stress-induced ligands like MICA/B and ULBPs on virus-infected or tumor cells, leading to NK cell activation, cytotoxicity, and cytokine production (e.g., IFN-γ and TNF-α). In contrast, inhibitory receptors such as killer cell immunoglobulin-like receptors (KIRs) and NKG2A bind to self-molecules like major histocompatibility molecule (MHC) class I, preventing uncontrolled NK cell responses and protecting healthy cells, a mechanism that tumors sometimes exploit by upregulating MHC class I to evade immune surveillance [4].

NK cell memory is a recently discovered phenomenon and is among one of the first instances of immunological memory identified within the innate immune system. Antigen-specific memory responses by NK cells were recognized over a decade ago in mice involving murine cytomegalovirus (MCMV) infection, where it was found that a subset of NK cells with Ly49H receptors could recognize the m157 viral protein on MCMV-infected cells. These Ly49H^+^ NK cells showed clonal proliferation, enhanced killing, and a long-term memory response. Around the same time, antigen-specific recall responses by NK cells were demonstrated in a Rag-deficient mouse model of hapten-induced contact hypersensitivity [5]. In this mouse model lacking T and B cells, contact hypersensitivity was induced by the adoptive transfer of hepatic NK cells from a mouse previously immunized with a chemical hapten like 2,4-dinitro-1-fluorobenzene (DNFB) [6]. A previous study also demonstrated that mouse splenic NK cells stimulated in vitro with interleukin-12 (IL-12), IL-15, and IL-18 acquired memory-like properties with enhanced IFN-γ production, which could be detected in Rag-deficient mice upon adoptive transfer [7]. However, unlike MCMV- or DNFB-induced memory NK cells, cytokine-activated NK cells show less pronounced cytotoxicity upon restimulation [8]. The establishment of memory in NK cells is a multifaceted process involving intricate signaling cascades, transcriptional regulatory mechanisms, and epigenetic modifications that collectively govern their memory-like functionality [9]. In the available scientific literature, these memory NK cell subsets are described using a diverse nomenclature, including adaptive NK cells, memory NK cells, memory-like NK cells, and antigen-specific NK cells, reflecting their functional and phenotypic heterogeneity [10]. Adoptive transfer into a NK cell-deficient host, followed by antigen challenge, showed that memory NK cells undergo rapid proliferation followed by a contraction phase, ultimately forming a small, stable population that persists for over six months within both lymphoid and non-lymphoid tissues [11]. Specifically, in the case of MCMV-specific Ly49H^+^ NK cells, these cells demonstrate prolonged longevity and retain their capacity for rapid proliferation upon subsequent MCMV challenge, mirroring their behavior observed in the context of MCMV infection. These findings suggest that homeostatic proliferation may generate NK cells with memory-like properties, though the underlying mechanisms remain poorly understood.

## 2. Hallmark of NK Cell Memory

### 2.1. Development and Key Features

**Figure 1:**
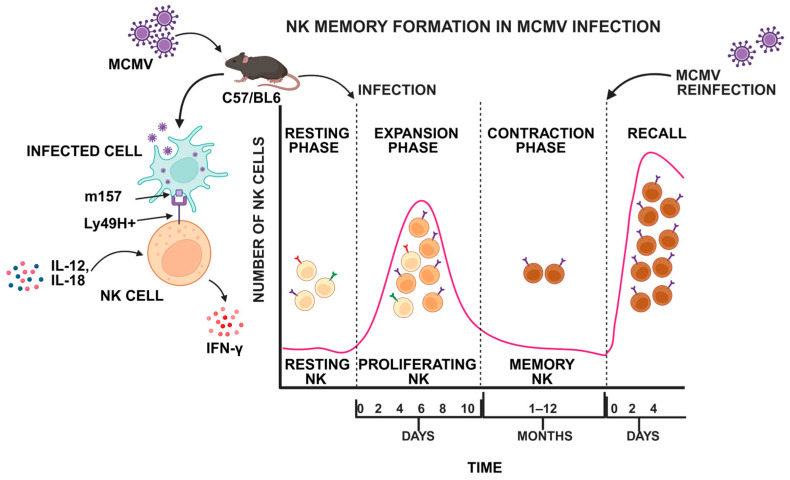
The NK cell response to MCMV infection follows a four-phase trajectory: (1) Resting NK cells are activated through the interaction between their activating receptor Ly49H and the viral ligand m157 on infected cells. (2) The activated NK cells produce IFN-γ, granzyme B, and perforin in response to IL-12 and IL-18 (days 1–3), followed by a rapid expansion of Ly49H^+^ NK cells (days 4–7). (3) Contraction and memory formation (days 7–30+), where a subset of NK cells persists, acquiring enhanced functional properties. (4) Upon MCMV reinfection, Ly49H^+^ NK cells exhibit an accelerated response, characterized by rapid proliferation, increased IFN-γ secretion, and heightened cytotoxic activity. The gradual color change in the proliferating and memory NK cells reflects their gradually altered gene expression and epigenetic modifications. Figure created in Biorender (https://www.biorender.com/ (accessed on 1 May 2025)).

When adaptive immune cells, like T cells, recognize their cognate antigen–MHC complex on antigen-presenting cells (APCs)—a process mediated by various cytokines, particularly IL-2—they undergo activation and clonal expansion, followed by a contraction phase in which 90–95% of the activated lymphocytes undergo apoptosis. The remaining 5–10% of T cells survive and differentiate into memory T cells, establishing a long-lived memory pool capable of mounting rapid and robust responses to subsequent antigen exposure [12]. The key features of this adaptive immunological memory include a rapid and amplified immune response upon re-exposure to antigens, enhanced antigen specificity due to affinity maturation, and the ability to provide long-lasting protection through the persistence of memory cells [13]. Unlike T and B cells, which undergo the RAG-mediated recombination of variable gene segments to generate a diverse array of antigen-specific receptors, NK cells lack RAG expression and rely on a limited repertoire of germline-encoded receptors with restricted antigen specificity [14]. Interestingly, in C57BL/6 mice infected with MCMV, Ly49H^+^ NK cells rapidly expand in response to the viral glycoprotein m157, and then contract to form memory-like NK cells, mirroring T cell dynamics. IL-12 and IL-18 are essential for their maintenance. Upon re-challenge, they show a reduced heterologous response but enhanced IFN-γ production (Figure 1) [15]. In addition to the evidence supporting NK cell memory formation during MCMV infection, several studies indicate the existence of memory-like NK cell responses against a diverse range of pathogens, including viruses such as Epstein–Barr virus (EBV), vaccinia virus, and human immunodeficiency virus (HIV); bacteria such as *Mycobacterium* sp.; and eukaryotic pathogens like *Plasmodium falciparum*; as well as autoimmune diseases such as type 1 diabetes mellitus (T1DM) and contact hypersensitivity. Moreover, memory-like NK cell responses have also been observed in certain cancers, including acute myeloid leukemia (AML) [16].

### 2.2. Tissue-Resident Memory NK Cells

In humans, NK cells originate in the bone marrow from CD34^+^ hematopoietic progenitor cells (HPCs) via an intermediate stage involving common lymphoid progenitors (CLPs) [17]. During this process, CLPs progressively acquire CD56 expression, a key surface marker that is characteristic of NK cells. Based on the expression of CD16 and CD56, NK cells are traditionally classified into two major subpopulations: NK1 (CD56^dim^CD16^+^), which are highly cytotoxic, and NK2 (CD56^bright^CD16^−^), which primarily have immunoregulatory functions [18]. In a recent study, the integration of single-cell RNA sequencing (scRNA-seq) with cellular indexing of transcriptomes and epitopes by sequencing (CITE-seq) data in healthy human populations identified three distinct subsets of NK cells: NK1, NK2, and NK3 [19]. These subsets correspond to canonical CD56^dim^ NK cells, canonical CD56^bright^ NK cells, and human cytomegalovirus (HCMV)-driven adaptive NK cells, respectively, with the NK1 and NK2 subsets predominantly found in blood and tissues such as the lungs, tonsils, and liver, while NK3 is enriched in gut and lung tissues [20]. In contrast, memory T cells are categorized into central memory T cells (T_CM_), primarily located in secondary lymphoid organs like lymph nodes and tonsils; effector memory T cells (T_EM_), which primarily inhabit non-lymphoid peripheral tissues such as the lungs, liver, and intestines and recirculate between blood and tissues; and tissue-resident memory T cells (T_RM_), which remain localized in specific tissues, including the digestive tract, lungs, skin, and brain, without recirculating [21]. However, the existence of a linear developmental subpopulation of NK memory cells has not yet been fully characterized. Adaptive-like CD49a^+^ CD56^bright^ NK cells with a tissue-resident phenotype are found in abundance in the lungs and the liver of HCMV-seropositive individuals [22]. In the lungs, they may arise from lung-tropic viral infections and show strong responsiveness and immunological potential [23], and in the liver, they exhibit stable phenotypes, terminal differentiation markers (KLRG1, NKG2C), reduced degranulation, and high cytokine production, suggesting a regulatory role [24].

In a hapten-specific contact hypersensitivity (CHS) model in mice, hapten-induced memory NK cells have been shown to localize specifically in the liver, with no significant presence observed in the spleen [25]. In contrast, Ly49H^+^ mouse memory NK cells, which are generated in response to MCMV, are predominantly found in the spleen, liver, and bone marrow, where they play a crucial role in systemic immune surveillance and prompt recall responses. Notably, MCMV-specific memory NK cells have also been identified in non-lymphoid tissues, such as the salivary glands and lungs, particularly during chronic infection [26]. This tissue-resident subpopulation of memory NK cells from the salivary glands exhibits a distinct tumor necrosis factor (TNF)-Related Apoptosis-Inducing Ligand (TRAIL^+)^ phenotype, and is recruited to the salivary glands through IFN-γ signaling in a CX3CR1-dependent manner [27]. During a vaccinia virus or *Staphylococcus aureus* infection in mouse models, Eomes^+^CD69^+^ tissue-resident memory NK (trNK) cells were identified in the skin, lacking CD49b, CD49a, CXCR6, and Ly6C2 [28]. These trNK cells undergo transcriptional changes compared to circulating NK cells and display stronger effector responses upon local re-challenge, indicating their role in enhanced immune protection. trNK cells heavily rely on transcription factors such as Tcf1, Eomes, and T-bet for their memory programming and effector functions. In contrast, tissue-specific NK cells, which reside in steady-state tissues, are more dependent on transcription factors like Hobit-, Runx3-, and tumor growth factor β (TGF-β)-dependent signaling, especially in organs like the liver and the uterus. Notably, Hobit is essential for establishing residency in steady-state NK cells but is not required for trNK cells, highlighting a significant transcriptional distinction between these two NK cell subsets [29].

## 3. NK Cell Memory in Viral Infection

### 3.1. DNA Viruses

#### 3.1.1. Cytomegalovirus

NK cell memory has been extensively studied in the context of cytomegalovirus infection models in both humans and mice. Studies on MCMV infection in C57BL/6 mice, which express the activating receptor Ly49H, have demonstrated that NK cells specifically recognize the viral glycoprotein m157, leading to robust clonal expansion and functional maturation. The immune response follows a distinct trajectory, beginning with the early activation phase, occurring within the first three days of infection, during which NK cells respond to inflammatory cytokines such as IL-12 and IL-18 by producing effector molecules like IFN-γ, granzyme B, and perforin [30]. This is followed by the expansion phase, between days four and seven, where Ly49H^+^ NK cells undergo significant proliferation, especially within the spleen, liver, and bloodstream. Subsequently, the response transitions into the contraction and memory formation phase, lasting beyond day seven, during which a subset of NK cells persists with heightened functionality. These memory-like NK cells exhibit an enhanced recall response upon secondary MCMV infection, characterized by rapid proliferation, increased IFN-γ production, and heightened cytotoxicity, akin to memory T cells (Figure 1) [31]. Studies indicate that these cells primarily reside in the bone marrow and liver, where they express CXCR6, facilitating their retention and mobilization upon reinfection [32]. IL-15 is essential for their long-term maintenance, as IL-15^−/−^ mice fail to sustain them, while adoptive transfer studies confirm their enhanced recall capacity [33].

The discovery of Ly49H^+^ memory NK cells in the MCMV infection model has sparked interests in investigating similar memory responses in human CMV infections. HCMV infection reshapes the NK cell receptor landscape, driving the expansion and long-term maintenance of memory-like NKG2C^+^KIR^+^ NK cells. Human leukocyte antigen (HLA) E peptides modulate NKG2C^+^ NK cell functionality, promoting the development of NKG2C^hi^ memory NK cells with elevated IL-7R and cyto-CD3ε expression [34]. Subgroups of adaptive HCMV-specific NK cells were also characterized by markers such as NKG2C, CD57, Foxo3, T-bet, and Zeb2, with reduced PLZF [35]. Among HCMV-seropositive individuals, it was found that adaptive NKG2C^+^ NK cells recognized distinct UL40 peptides from HCMV strains, which, along with pro-inflammatory signals, drove NK cell proliferation and differentiation, shaping their heterogeneity [36]. The interaction between CD2 and CD58 is critical for the activation and functional efficacy of adaptive NKG2C^+^CD57^+^ NK cells in the context of HCMV infection [20]. Additionally, three novel HLA-E-restricted 15-mer peptides from the HCMV pp65 protein were identified, eliciting specific adaptive NK cell memory responses to HCMV [37].

#### 3.1.2. Epstein–Barr Virus

NK cells play a critical role in controlling Epstein–Barr Virus (EBV)-induced B cell transformation and associated pathologies, including Burkitt’s lymphoma, Hodgkin’s lymphoma, and post-transplant lymphoproliferative disorders (PTLDs) [38]. In contrast to HCMV infection, acute EBV infection does not promote the expansion of NKG2C^hi^ memory-like NK cells, but instead leads to an increased frequency of mature CD56^dim^NKG2A^+^KIR^−^CD57^+^ NK cells that persist for at least 2.5 years [39]. This CD56^dim^NKG2A^+^KIR^−^CD57^+^ NK cell subset characterized by an early-differentiated phenotype accumulated in the secondary lymphoid organs, like the tonsils of EBV^+^ individuals. Activation, these cells exhibit a heightened ability to release IFN-γ and efficiently restrict EBV infection and B-cell tumorigenesis. [40]. During the latency phase of EBV infection, infected cells evade NK cell recognition; however, upon reactivation or oncogenic transformation, they become more susceptible to NK cell-mediated cytotoxicity due to the downregulation of HLA-I and upregulation of stress-inducible proteins [41]. In a comparable manner, it has been demonstrated that NKG2A^+^ NK cells exhibit prolonged persistence at heightened frequencies within the bloodstream of humanized mice for several weeks subsequent to infection with EBV [38]. The memory-like functions of these expanded NKG2A^+^ NK cells remain unexplored, and further investigation is needed to understand their phenotypic changes, their suitability for targeting EBV-infected cells across viral expression programs, and their underlying molecular mechanisms.

#### 3.1.3. Herpesvirus

Studies on the human alphaherpesvirus family have demonstrated their capacity to induce adaptive NK memory responses. Varicella zoster virus (VZV), a prominent member of this family and the etiological agent of chickenpox and shingles, has been demonstrated to elicit pronounced delayed-type hypersensitivity (DTH) reactions following the intradermal administration of VZV antigens in individuals with a prior history of childhood chickenpox [20]. Importantly, the recruited NK cells predominantly exhibited a CD56^high^ phenotype and expressed tissue-residency-associated markers such as CXCR6, NKG2D, CD69, and CD62L [42]. However, the contributions of T and B lymphocytes to these responses could not be definitively excluded due to the inherent limitations of the human study design. Research on another prevalent alphaherpesvirus, herpes simplex virus type 2 (HSV-2), using Rag1^−/−^ mice lacking B and T cells, demonstrated that prior infection with an attenuated HSV-2 strain conferred resistance to a subsequent lethal HSV-2 re-challenge. Resistance to secondary HSV-2 infection depended on memory-like NK cells, as their depletion negated protection, and NK cells from HSV-2-primed mice showed transient (~30 days) enhanced IFN-γ production, unlike the prolonged memory seen in MCMV infections [43]. Thy1^+^ (CD90^+^) NK cells mediate memory responses against vaccinia virus, a DNA virus from the Poxviridae family, enabling its clearance independent of adaptive T and B lymphocytes [44]. Memory NK cells, especially in hepatic tissues, depend on CXCR6 signaling via CXCL16 produced by sinusoidal epithelium, with markers such as CD49a and Hopx being upregulated during vaccinia virus (VACV) infection, linking them to memory and effector functions [45].

### 3.2. RNA Viruses

#### 3.2.1. Human Immunodeficiency Virus

Like DNA viruses, a lot of RNA viruses have been found to provoke a memory response in NK cells. Innate immune responses, particularly those of NK cells, play a crucial role in the early control of HIV-1 infection and the development of adaptive immunity. Studies show that NK cell numbers increase during the hyperacute phase (Fiebig stages I and II) during acute HIV infection (AHI), dominated by CD56^dim^ cytotoxic NK cells, while CD56^bright^ NK cells decline [46]. South African cohort genes linked to NK cytotoxicity and chemokine signaling were upregulated around peak viremia, with NK cell plasticity declining over time. The presence of proliferative and cytotoxic NK cells in early infection is associated with long-term viral control and higher CD4^+^ T cell counts for years without treatment [47]. In HIV-1 cohorts, HCMV is the most common coinfection, driving the reconfiguration of the well-characterized CD57^+^NKG2C^+^ adaptive NK cell with enhanced CD16-mediated activation and antibody-dependent cellular cytotoxicity (ADCC) responses to HIV-1 peptides. The absence of PLZF serves as a definitive biomarker that distinguishes CD57^+^NKG2C^+^ adaptive NK cells from other NK cell subsets. In HIV-positive individuals, PLZF-deficient NK cells exhibited elevated CD2 expression, a lack of Siglec-7, and a marked downregulation of key signaling molecules, including SYK and FcεRI-γ [48]. These cells predominantly displayed an adaptive NK cell phenotype, which was significantly associated with higher serum antibody titers against HCMV. Despite the limitations of human studies and the challenge of characterizing HIV-specific adaptive NK cells due to HCMV coinfection, CMV-primed CD57^+^NKG2C^+^ adaptive NK cells have been shown to inversely correlate with viral load, highlighting the critical role of memory NK cells in early HIV-1 infection [49]. With Modified Vaccinia virus Ankara (MVA) vaccination, NK cells exhibit a transcriptional shift marked by the upregulation of genes associated with cell cycle progression and cytotoxic activity, indicating enhanced memory-like NK cell responsiveness against HIV-1 [50]. Mechanistic evidence of human NK cell HIV-specific memory has been linked to elite controllers, with responses dependent on NKG2C-HLA-E interactions involving HIV-1 Gag and Env peptides [51]. Transcriptomic and phenotypic analyses reveal that these antigen-specific NK cells co-express NKG2C, KLRG1 (also implicated in HBV-specific NK responses), and α4β7, suggesting mucosal homing potential [52]. It has been evaluated that the HIV latency-reversing agent (LRA), 3-hydroxy-1,2,3-benzotriazin-4(3H)-one (HODHBt), can induce memory-like functions in NK cells [53]. HODHBt enhances the STAT signaling pathway, leading to the activation of IL-15-induced memory NK cells with an increased production of CXCL-10 and IFN-γ, along with an upregulation of cytotoxic proteins such as granzyme B, granzyme A, perforin, granulysin, FASL, and TRAIL [53].

#### 3.2.2. Influenza Virus

Influenza, a highly contagious RNA virus, remains a significant global health concern due to its potential to cause widespread outbreaks and severe respiratory complications. Studies on influenza-vaccinated individuals have shown that, three months post-vaccination, blood NK cells exhibit memory NK cell-like features. These NK cells were found to show increased IFN-γ and superior degranulation upon in vitro restimulation with inactivated influenza alongside a low dose of IL-12 and IL-18, with this augmented functionality being partly reliant on type I interferon signaling [54]. Recent studies have shown that influenza vaccines stimulated memory NK cells characterized by intracellular NKp46 expression, where the internalization of surface NKp46 and the progressive rise in NKp46 (intracellular)^+^ CD56^dim^ NK cells were positively associated with enhanced IFN-γ production upon influenza virus restimulation post-vaccination [55]. Intracellular NKp46 expression correlates positively with IFN-γ responses, suggesting its crucial role in the NK cell recall response to influenza antigens. In a mouse model, a novel hemagglutinin (HA)-specific NKp46^+^ NKG2A^+^ NK cell subset was identified through intranasal influenza virus infection [56]. These memory NK cells were recruited to the infected lung, where they facilitated viral clearance and influenced CD8^+^ T cell distribution, ultimately leading to improved clinical outcomes upon re-exposure to the same influenza virus antigen [57]. Additionally, other studies have identified memory-like liver NK cells, characterized by a CD49a^+^DX5^−^ phenotype, which provide protective immunity and reduce viral load when adoptively transferred into naive mice which were subsequently exposed to viral infection [58]. Furthermore, the primary inactivated influenza virus has been shown to elicit memory NK cells in the liver of Rag^−/−^ mice, highlighting their potential role in adaptive immune responses [59].

#### 3.2.3. Severe Acute Respiratory Syndrome Coronavirus 2

Severe Acute Respiratory Syndrome Coronavirus 2 (SARS-CoV-2) is an enveloped, positive-sense, single-stranded RNA virus responsible for the global COVID-19 (corona virus disease 2019) pandemic, exhibiting high transmissibility and immune evasion strategies that modulate host immunity. Recent evidence suggests that SARS-CoV-2 infection induces a subset of memory-like NK cells, contributing to long-term antiviral immunity. Studies have identified an expansion of NKG2C^+^CD57^+^FcεRIγ^−^ memory NK cells in COVID-19 patients, particularly in individuals with prior HCMV infection, suggesting potential cross-reactivity and enhanced antiviral effects [60]. Pro-inflammatory cytokine signaling upregulates HLA-E expression, augmenting NKG2C^+^CD57^+^ NK cell differentiation [61]. SARS-CoV-2 infection and vaccination induce significant alterations in NK cell differentiation and functional plasticity in people living with HIV (PLWH), influenced by chronic immune activation and CD4^+^ T cell depletion [62]. SARS-CoV-2-naive PLWH exhibit the induction of adaptive NK cells, alongside a distinct CD56^bright^ NK cell population with enhanced cytotoxic potential post-vaccination [63]. Antibody-dependent NK cell responses demonstrated robust and durable spike-specific cytotoxicity up to 148 days postinfection, enriched within the adaptive NK cell pool. NK cell-mediated immunity was further boosted by the first vaccine dose in SARS-CoV-2-exposed individuals, with responses peaking post-second dose in naive PLWH. The magnitude of adaptive NK cell expansion correlated with the strength of both cellular and humoral immunity, reinforcing their role in long-term immune protection. These findings highlight the potential of memory NK cells as key effectors in enhancing vaccine-induced immunity, particularly in vulnerable populations such as PLWH [64].

#### 3.2.4. Flavivirus

Zika virus (ZIKV) is a mosquito-borne flavivirus known for causing mild febrile illness in most cases, with associated severe neurological complications, including microcephaly in newborns and Guillain–Barré syndrome in adults. A study in a mouse ZIKV infection model described a distinct population of TCF1^hi^ CD27^+^ NK cells, called “NK memory stem cells”, which showed increased antiviral activity compared to their CD27^−^ or naive CD27^+^ counterparts [65]. A study also found out that the early activation of NK cells postinfection was characterized by an increase in CD27^+^KLRG1^+^ NK cells that produced elevated levels of IFN-γ and perforin, which normalized one month later; however, the examination of CD27^+^ NK cells at that time revealed a memory-like phenotype [65]. Analysis of blood from ZIKV-infected individuals shows significant NK cell activation, while murine NK cells that acquire memory-like properties following in vivo ZIKV infection exhibit enhanced antiviral efficacy.

Patients infected with hepatitis C virus (HCV) show an increased presence of mature NK cell phenotypes (CD16, KIR, CD57, and KLRG1) within CD56^dim^ NK cells, exhibiting enhanced degranulation against in vitro cells infected with various HCV strains [66]. This antigen-specific NK cell response persists after viral clearance and operates independently of T cells and IL-2, indicating a recall memory response upon re-exposure to HCV-infected cells [66].

#### 3.2.5. Hantavirus

In hantavirus-infected patients, a significant expansion of CD56^dim+^ NKG2C^+^ NK cells was observed, with proliferation persisting for over 60 days, indicating potential memory-like properties in human NK cells. Elevated plasma IL-15 levels in these patients correlated positively with NKG2C^+^ NK cell expansion, suggesting a role in their activation and maintenance [67]. The increased frequency of NKG2C^+^ NK cells was not associated with CMV reactivation but may be attributed to elevated plasma IL-15 levels, which serve as a key driver of NKG2C^+^ NK cell expansion [67,68]. This suggests a crucial role for IL-15 in their activation and sustained maintenance, indicating an alternative mechanism underlying their proliferation.

## 4. Molecular Mechanisms of Virus-Induced NK Cell Memory Formation

NK cells exhibit memory-like characteristics upon infection with various viruses. The molecular basis of this immunological memory primarily involves receptor signaling, cytokine-mediated priming, epigenetic reprogramming, and metabolic shifts, which are highly interconnected and often exhibit cause-and-effect relationships.

### 4.1. Receptor Signaling

**Figure 2:**
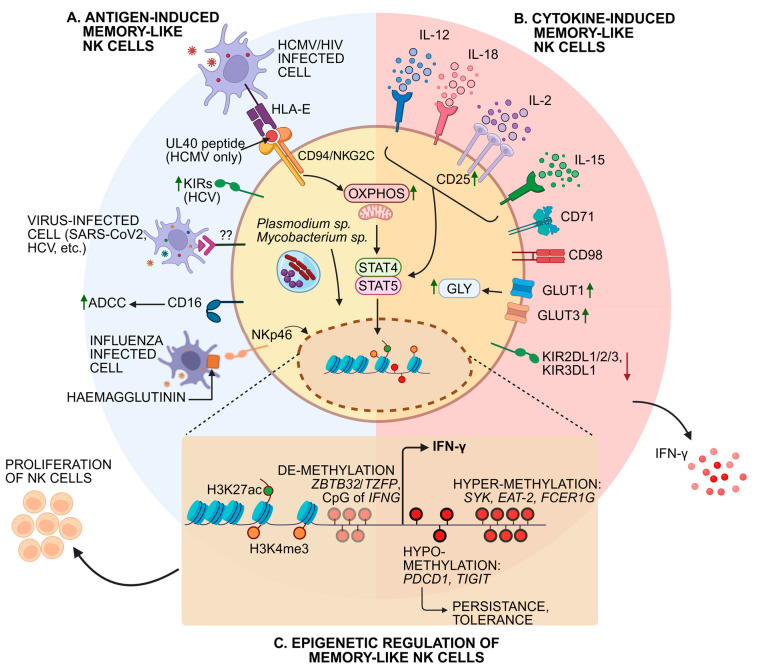
Mechanisms of human NK memory cell generation: (**A**) Antigen-induced memory-like NK cells (left, blue section): These cells develop in response to viral infections (e.g., HCMV, HIV, influenza) and intracellular pathogens (e.g., *Plasmodium* sp., *Mycobacterium* sp.). Key mechanisms include CD94/NKG2C interaction with HLA-E, upregulation of killer cell immunoglobulin-like receptors (KIRs), and activation through CD16-mediated antibody-dependent cellular cytotoxicity (ADCC). (**B**) Cytokine-induced memory-like NK cells (right, red section): Cells generated following exposure to cytokines such as IL-12, IL-18, IL-2, and IL-15. Cytokine stimulation enhances metabolic activity like OXPHOS and Gly (glycolysis), increases activation markers (CD25, CD71, CD98), and upregulates glucose transporters (GLUT1, GLUT3). Additionally, it also influences expression of specific KIRs. (**C**) Epigenetic and functional modifications (bottom panel): Under both antigen- and cytokine-induced conditions, memory-like NK cells undergo epigenetic modifications. These include demethylation of IFN-γ regulatory regions (ZBTB32/TFZF) and selective hypo-/hyper-methylation of genes associated with persistence (PDCD1, TIGIT) and immune signaling (SYK, EAT-2, FcεRI-γ), contributing to enhanced NK cell longevity and function. Green upward-pointing arrows indicate upregulation, while red downward-pointing arrows represent downregulation. Question marks denote unknown or unconfirmed receptor-ligand interactions. Figure created in with Biorenderer (https://www.biorender.com/ (accessed on 1 May 2025)).

Ly49 receptors in mice recognize MHC-I and MHC-I-like proteins on both normal and altered cells, with Ly49D/H activating memory NK cell responses through DAP12/DAP10 signaling. Similarly, in humans, KIRs play a crucial role in NK cell education, regulating activation and inhibition to maintain immune balance. The NKR-P1C (NK1.1) receptor binds the m12 glycoprotein of MCMV, facilitating memory formation in liver-resident ILC1s, which undergo epigenetic modifications postinfection [69]. Similarly, HCMV-driven adaptive NK cells exhibit a high expression of NKG2C/CD94, enabling the recognition of HLA-E presenting UL40 peptides, leading to *IFNG* locus hypomethylation and heightened responsiveness [20]. However, chronic NKG2C-mediated activation drives exhaustion-like states, marked by the hypomethylation of immune checkpoint genes such as PDCD1 and TIGIT, resembling T cell dysfunction. CD16 (FcγRIII) mediates ADCC, with preactivation enhancing NK cell responses akin to cytokine-induced memory [70]. CD16 undergoes transient downregulation postactivation, but remains essential for sustained ADCC function. FcεRIγ-deficient NK cells (FcεRIγ−) exhibit enhanced ADCC, indicating a specialized adaptation [71]. DNAM-1 (CD226) plays a critical role in MCMV-driven NK memory, signaling through GRB2 and PKC-η, while Fyn kinase supports late-stage memory differentiation [72]. The inhibition of CD226 signaling reduces FOXO1 phosphorylation, impairing NK cell proliferation and function. Additionally, CD2 costimulation synergizes with NKG2C, further augmenting IFN-γ production in adaptive NK cells (Figure 2A) [73].

### 4.2. Cytokine Signaling

Acute MCMV infection induces a robust pro-inflammatory cytokine response, including IL-12, IL-18, and type I interferons (IFNs) [10]. These cytokines are crucial in driving NK cell proliferation and differentiation during the expansion phase of memory formation. IL-12, IL-15, and IL-18 act synergistically to induce CD25 expression, leading to the formation of a high-affinity IL-2 receptor that enhances NK cell proliferation and antiviral IFN-γ production [74]. Cytokine responses vary across viral infections. IL-12 plays a potent antiviral role against HSV-2, inducing IFN-γ and protecting mice from lethal infection [75]. IL-18 is differentially expressed during influenza virus and HIV-1 infections, and SARS-CoV-2-induced IL-12, IL-18, and IL-15 secretion drive adaptive NK cell differentiation. IL-12 and IL-18 are also critical for controlling HBV and HCV infections [76]. Additionally, IL-15-mediated JAK/STAT signaling is essential for memory NK cell homeostasis, survival, and proliferation, with elevated CD122 (IL-2Rβ) expression enhancing IL-15 responsiveness (Figure 2B) [77].

### 4.3. Epigenetic Reprogramming and Transcriptional Regulation

Adaptive and memory-like NK cells undergo extensive epigenetic remodeling, resulting in stable transcriptional changes that enhance recall responses. Histone modifications, such as to H3K4me3 and H3K27ac, at NK cell effector gene loci promote sustained transcriptional activation [78]. In HCMV infections, memory NK cells exhibit epigenetic modifications, including the hypermethylation of genes encoding key activating receptors; signaling molecules such as the tyrosine kinase SYK, adaptor proteins EAT-2, and FcεRIγ; and the transcription factor PLZF (Figure 2C) [72]. These modifications, also observed in influenza virus infections, contribute to the functional adaptation of NK cells. FcRγ-deficient NK cells, which display enhanced antibody-dependent functional activity, often show a reduced expression of these signaling proteins due to epigenetic silencing [79]. Furthermore, the demethylation of the PRDM1/BLIMP1 and ZBTB32/TZFP genes plays a role in the long-term persistence of memory NK cells [80]. The CpG demethylation of the *IFNG* locus increases IFN-γ production and facilitates cytokine-induced memory-like NK cell survival and cytotoxicity, while hypermethylation of the *FCER1G* gene is commonly detected in NKG2C^+^ NK cells, a hallmark of adaptive NK cell responses [80].

### 4.4. Metabolic Adaptations for Long-Term NK Cell Survival

**Figure 3:**
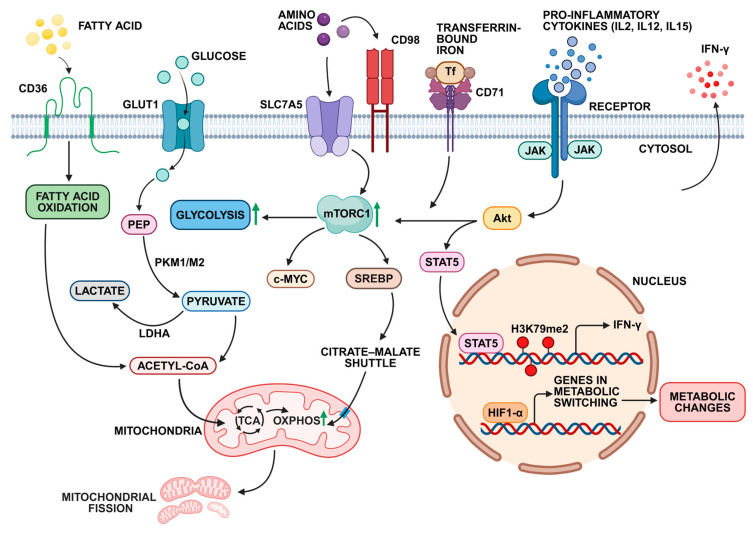
The metabolic reprogramming pathways in memory NK cells derived from murine and human studies: GLUT1 is the primary glucose transporter upregulated in memory NK cells, enhancing glucose uptake and oxidative phosphorylation (OXPHOS). mTORC1 is a central regulator of NK cell metabolism, promoting glycolysis and OXPHOS while also activating cMYC and SREBP, which further modulates these pathways. The amino acid transporter SLC7A5 is crucial for sustaining cMYC and mTORC1 signaling, supporting energy production and biosynthesis. The transferrin receptor CD71, a cMYC target, facilitates iron uptake, essential for enzymes in mitochondrial electron transport. IL-2 and IL-12 stimulation enhance cMYC expression via mTORC1, which also regulates SREBP activation. Cytokines induce SLC7A5 expression, supporting NK cell metabolism, while SREBP regulates glucose metabolism through the citrate–malate shuttle. HIF-1α signaling regulates NK cell metabolism by promoting glycolysis and enhancing mitochondrial fission and fitness through Drp1 activation. Cytokines like IL-15 activate the PI3K/AKT pathway, leading to mTORC1 activation, epigenetic modifications, and IFN-γ induction, further shaping NK cell function. Green upward pointing arrow indicates upregulation. Figure created in with Biorenderer (https://www.biorender.com/ (accessed on 1 May 2025)).

Virus-induced memory NK cells undergo metabolic reprogramming to sustain their longevity and function. Unlike conventional NK cells, which primarily depend on glycolysis, memory NK cells favor oxidative phosphorylation (OXPHOS) and mitochondrial biogenesis, enhancing their survival and responsiveness to reinfection [81]. During MCMV infection, splenic NK cells initially upregulate glycolysis and the pentose phosphate pathway to meet early activation energy demands [82]. However, as the infection progresses, mitochondrial remodeling occurs, with the early accumulation of dysfunctional mitochondria and mitochondrial reactive oxygen species (mtROS), which are later resolved as NK cells transition to a memory phenotype [83]. In murine models, glycolysis plays a crucial role in antiviral immune responses. The pharmacological inhibition of glycolysis using 2-deoxyglucose (2-DG) impairs NK cell-mediated cytotoxicity; however, this defect can be rescued by IL-15 stimulation [33]. Interestingly, the genetic deletion of pyruvate kinase M2 (PKM2) in mice does not significantly affect NK cell responses due to compensatory PKM1 expression [84]. However, viral infection in NK cell-specific lactate dehydrogenase A (LDHA) knockout mice is exacerbated, highlighting the critical role of aerobic glycolysis in antiviral immunity [85]. Studies using an NK cell-specific mechanistic target of rapamycin com-plex 1 (mTORC1) knockout mice have shown that mTORC1 regulates NK cell metabolism in an IL-15-dependent manner, primarily influencing early activation responses while having minimal impact on later effector functions [86]. In human HCMV-specific NKG2C^+^, cytokine-induced memory-like (CIML) NK cells undergo stable chromatin remodeling, promoting metabolic reprogramming toward enhanced glycolysis and mitochondrial biogenesis (see Section 4.3 and Section 8) [87]. Hypoxia-inducible factor 1-alpha (HIF1α) promotes glycolysis while modulating early activation [88], while mitochondrial function plays a crucial role in antigen-driven NK cell responses, with OXPHOS supporting memory formation and long-term survival (Figure 3) [89]. Additionally, memory-like NKs in latent tuberculosis infection (LTBI) exhibit upregulated CD226 expression following γ-Mtb stimulation [90]. The conditional deletion of Cox10, essential for the electron transport chain, disrupts NK cell expansion, highlighting OXPHOS’s significance [91]. Memory NK cells rely on fatty acid oxidation (FAO), especially in tissue-resident ILC1s [92]. HCMV-specific adaptive NK cells exhibit enhanced metabolic activity favoring amino acid metabolism over glucose. Increased c-Myc expression, linked to amino acid transport via SLC7A5, supports metabolic adaptation [93]. Overall, these metabolic shifts sustain NK cell memory and provide potential targets for enhancing antiviral immunity.

## 5. NK Cell Memory in Bacterial and Parasitic Infection

### 5.1. Mycobacterium Tuberculosis

Recent studies have demonstrated the presence of memory-like NK cell responses against *Mycobacterium tuberculosis* (Mtb). In active tuberculosis (TB) patients, CD45RO^+^ NK cells, a hallmark of memory-like properties, were identified in pleural fluid. Upon stimulation with interleukins and *Mycobacterium bovis* BCG (Bacillus Calmette–Guérin), these cells exhibited significantly elevated IFN-γ and IL-22 production compared to CD45RO^−^ NK cells [94]. In murine models, BCG vaccination induced an IL-21-dependent expansion of a distinct CD3^−^NKp46^+^CD27^+^KLRG1^+^ NK subset in the lymph nodes and spleen [95]. These cells conferred protection against Mtb upon adoptive transfer into naive mice, underscoring their role in host defense. IL-21, a pivotal cytokine in NK cell regulation, enhances the proliferation and effector function of NK cells in response to Mtb, as observed in LTBI, where Mtb-specific CD4^+^ and NKT cells secrete IL-21 to augment NK cell-mediated monocyte lysis and bacterial containment [95]. CD226 signaling was identified as a critical regulator of memory NK responses, as CD226 blockade via antibodies or CRISPR/Cas9 led to impaired proliferation, FOXO1 phosphorylation, and cMyc expression, with cMyc inhibition further suppressing memory NK proliferation [96]. Moreover, CD226 blockade reduced glycolysis, a metabolic process essential for memory NK effector functions [97]. Furthermore, the stimulation of CD45RO^+^ NK cells from pleural fluid cells (PFCs) with IL-12 resulted in enhanced IFN-γ production and cytotoxic activity compared to CD45RO^−^ NK cells [94]. IL-12 stimulation consistently upregulated the expression of granzyme B, CD69, CD25, NKG2D, and IL-12 receptors (β1 and β2) on CD45RO^+^ NK cells, reinforcing their role in adaptive-like immune responses against Mtb [98].

### 5.2. Ehrlichia sp.

In a C57BL/6 mice model of ehrlichiosis, a disease related to Rickettsial infections, primary infection with a non-virulent stain (*Ehrlichia muris*) shows long-lasting immunity against subsequent exposure to the virulent strain (*Ixodes ovatus Ehrlichia*), which is otherwise fatal. The depletion of NK cells in *E. muris*-primed mice abolished this protective memory response, leading to 80% mortality within 8–10 days post-IOE infection, similar to naive mice, highlighting the essential role of memory NK cells in *Ehrlichia*-induced immunity [99].

### 5.3. Salmonella Typhi

In the case of typhoid fever, the transcriptional profiling of individuals vaccinated with live-attenuated typhoid vaccines has demonstrated a strong correlation with the activation of cytokine-secreting memory-like NK cells, as confirmed through peripheral blood mononuclear cell (PBMC) stimulation. Ty21a-induced memory NK cells play a pivotal role in vaccine-mediated immunity by enhancing early IFN-γ production, thereby augmenting antibacterial defense mechanisms. Furthermore, CD226 signaling has been identified as a critical regulator of Ty21a-induced memory NK cell proliferation and effector function [100]. The inhibition of CD226 through targeted blockade results in reduced FOXO1 phosphorylation, cMyc expression, and glycolytic activity, collectively impairing NK cell-mediated immune responses [101].

### 5.4. Plasmodium sp.

Recent investigations utilizing murine models of *Plasmodium yoelii*, *Plasmodium berghei*, and human malaria infections have identified a subset of NKG2C^+^ CXCR6^+^ CD57^+^ NK cells with memory-like properties [102]. Upon re-challenge with the same *Plasmodium* sp. strain, these memory NK cells exhibited not only enhanced IFN-γ production and increased cytotoxic activity but also significantly augmented ADCC. Studies conducted in malaria-endemic regions of Mali using multi-parameter flow cytometry revealed a high frequency of adaptive NK cells, characterized by the downregulation of the transcription factor PLZF and the Fc receptor γ-chain [103]. These adaptive NK cells were the predominant mediators of ADCC responses, and their relative abundance within the total NK cell population demonstrated a strong correlation with reduced parasitemia and increased resistance to malaria infection.

## 6. NK Cell Memory in Immunological Disorders

A study identified a distinct subset of memory-like CD3^−^CD8^dull^CD56^+^ NK cells that exhibited reactivity to the glutamic acid decarboxylase 65 (GAD65) AA 114–122 peptide in individuals newly diagnosed with T1DM. The interaction of the GAD65 AA 114–122 peptide with the ILT2 inhibitory receptor induces NK cell proliferation and enhances CD107a expression or degranulation. However, the pathological relevance of this CD3^−^CD8^dull^CD56^+^ memory-like NK cell subset, particularly its heightened responsiveness upon secondary antigenic challenge in T1DM, remains to be elucidated [104]. In rheumatoid arthritis (RA), CIML NK cells exhibit impaired cytotoxicity upon restimulation and reduced IFN-γ production, potentially due to altered NKp46 and CD158e receptor expression [105]. This dysfunction may indicate their involvement in the memory response underlying RA pathogenesis. NK cells with memory-like properties are hypothesized to play a crucial role in sustaining prolonged remission. The presence of CD8^+^CD57^+^ NK cells in individuals with stable remission suggests their potential contribution to immune homeostasis and the mitigation of disease flare-ups [106]. Hapten-specific memory Ly49C-I^+^ NK cells are primarily localized within the hepatic microenvironment [107]. These memory NK cells mediate CHS responses in immunologically naive mice, demonstrating their capacity for antigen-specific recall. Experimental models of CHS in mice highlight NK cells’ role in sustaining antigen-specific memory, as CHS persisted in immunocompromised RAG2-deficient or severe combined immunodeficiency (SCID) mice lacking T and B cells [108]. However, depletion of NK cells alone or in combination with adaptive lymphocytes abolished the response, underscoring NK cells’ ability to facilitate adaptive recall responses independently of conventional lymphocytes.

## 7. NK Cell Memory and Cancer

The interaction between NK cells and cancer is complex. Initially, tumor cells can stimulate NK cell activation, leading to effective tumor killing. However, persistent antigen exposure and an immunosuppressive tumor microenvironment (TME) can lead to NK cell exhaustion or reduced activity [109]. Similarly to the sensitization of CD8^+^ T cells through pre-exposure to cancer cells, which leads to the development of antigen-specific memory, researchers have explored memory-like responses in NK cells [59]. This was achieved by priming NK cells with irradiated cancer cells, followed by a re-challenge with the same target cancer cells. These tumor-induced memory-like (TIML) NK cells demonstrated enhanced tumor-specific cytotoxicity compared to non-primed NK cells or those primed with irradiated PBMCs [110]. These findings were observed in studies using a pediatric B cell precursor acute lymphoblastic leukemia (BCP-ALL) cell line (NALM-16), as well as primary BCP-ALL and AML samples [111]. In contrast to CIML NK cells, TIML-NK cells displayed a heightened antigen specificity, an absence of upregulated CD25 expression, increased perforin release relative to IFN-γ production, and significant alterations in the expression of genes associated with metabolic pathways [112]. The unique characteristics of TIML-NK cells, along with their similarities to CIML-NK cells, distinguish them from HCMV-induced adaptive NK cells [113]. Notably, the precise phenotypic markers and the specific ligands or antigens responsible for driving the clonal expansion of TIML-NK and CIML-NK cells remain poorly understood. Human TIML NK cells exhibit an enhanced secretion of IFN-γ and TNF, alongside both immediate and sustained cytotoxic activity against head and neck squamous cell carcinoma (HNSCC) cell lines and primary tumor targets, surpassing the functional capacity of conventional NK cells [114]. Notably, memory-like NK cells display elevated NKG2A expression, a trait correlated with patient response metrics, highlighting its pivotal role in tumor recognition and immune modulation [115]. NKG2A, in conjunction with other activating receptors such as NKG2C, facilitates NK cell-mediated cytokine release and degranulation upon engagement with melanoma cells, thereby augmenting antitumor immunity [116]. Additionally, metabolic reprogramming via LDHA is critical for NK cell-mediated tumor cytotoxicity. In the tumor microenvironment, LDHA-driven aerobic glycolysis is indispensable for NK cell activation and effector functions against malignant cells. Beyond its role in cancer immunity, LDHA is also essential for the clonal expansion and memory formation of NK cells, as demonstrated in MCMV infection models, further underscoring its significance in NK cell-mediated immune responses [82].

## 8. Cytokine-Induced Memory NK Cells

NK cells can develop memory-like properties following cytokine exposure, exhibiting enhanced cytotoxicity compared to naive NK cells. Studies in both murine and human models demonstrate that prestimulation with IL-12 and IL-18 synergistically induces IFN-γ production and promotes a memory-like phenotype, while the addition of IL-15 further enhances survival and proliferative persistence [117]. Upon secondary challenge, these CIML-NK cells display increased cytotoxicity, elevated IFN-γ and TNF-α production, and prolonged survival. CIML-NK cells exhibit distinct phenotypic alterations, including the transient downregulation of CD16 without compromising ADCC, followed by CD16 restoration upon cytokine reactivation [118]. Additionally, these cells demonstrate sustained proliferation in low IL-2 concentrations due to their upregulated expression of the high-affinity IL-2 receptor CD25 [119]. Further, IL-12/15/18-activated CIML-NK cells show a reduced expression of inhibitory receptors (KIR2DL1, KIR2DL2/L3, KIR3DL1), which is reversible upon IL-2 restimulation. Beyond surface marker modulation, CIML-NK cells undergo metabolic reprogramming, characterized by the increased expression of nutrient transporters (CD71, CD98) and glucose uptake receptors (GLUT1, GLUT3) [120]. Enhanced ATP production via glycolysis and oxidative phosphorylation supports their effector functions, as the inhibition of these pathways post-cytokine-stimulation diminishes IFN-γ release [121]. Unlike adaptive immune memory, which relies on genetic recombination, CIML-NK cell memory is driven by epigenetic and metabolic adaptations. IL-12/15/18 stimulation activates STAT signaling pathways, leading to the demethylation of the IFN-γ CNS1 region in both murine and human models (Figure 3) [122]. Furthermore, the histone methyltransferase EZH2 plays a critical role in regulating IFN-γ production upon NK cell reactivation by IL-12 and IL-18 [123].

## 9. Molecular Mechanisms of Antigen-Independent NK Cell Memory Formation

Unlike virus- or antigen-induced NK cell memory, CIML NK cells primarily rely on pro-inflammatory cytokine signaling, particularly IL-12, IL-15, and IL-18 [124]. This cytokine-driven activation not only enhances their longevity and cytotoxic function but also induces significant epigenetic and metabolic reprogramming. IL-12, IL-15, and IL-18 activate STAT4 and STAT5, leading to the upregulation of Blimp1 (Prdm1) and the downregulation of Bcl6, which together promote the differentiation of effector CIML NK cells and drive metabolic rewiring, characterized by increased OXPHOS and enhanced glycolytic flux, facilitated by HIF-1α [125]. In addition, strong cytokine signaling drives epigenetic modifications that reinforce memory-like properties. CIML NK cells exhibit transient epigenetic modifications, including reversible DNA methylation, enhancing immediate responsiveness. Both CIML and virus-induced NK memory cells undergo *IFNG* locus demethylation, promoting IFN-γ production [80]. In contrast, virus-induced NK memory cells undergo stable epigenetic reprogramming, characterized by persistent DNA demethylation at *IFNG* and *ZBTB32*, and chromatin remodeling that sustains their long-term memory-like functionality [126].

## 10. NK Cell Memory in Immunotherapy Development

Utilizing the memory-like phenotype of NK cells in adoptive cellular therapy has gained significant traction in recent years due to their enhanced persistence, recall response, and superior cytotoxic potential. TIML-NK cells and CIML-NK cells exhibit distinct activation mechanisms and demonstrate differential responses upon secondary antigenic stimulation [114]. While tumor-primed NK cells display augmented direct cytotoxicity against malignant cells, CIML-NK cells exhibit enhanced cytokine secretion, like IFN-γ, prolonged survival, and increased proliferative capacity, making them particularly effective in relapsed/refractory hematological malignancies [118]. The therapeutic relevance of CIML-NK cells was highlighted first in human clinical trials, where the adoptive transfer of cytokine-preactivated NK cells in AML patients demonstrated both safety and clinical efficacy. Reports indicate that CIML-NK therapy resulted in complete remission (CR) in 47% of treated patients, while notably lacking severe adverse events such as cytokine release syndrome (CRS), neurotoxicity, or graft-versus-host disease (GvHD) [127]. Moreover, CIML-NK cells exhibited a robust graft-versus-leukemia (GvL) effect, successfully targeting leukemic cells in relapsed/refractory AML patients who had undergone prior transplantation with cytokine-activated NK cells. These promising results have led to ongoing clinical trials evaluating CIML-NK cells in various malignancies, including AML, myelodysplastic syndromes (MDSs), multiple myeloma (MM), and HNSCC [128]. Additionally, KIR^+^NKG2C^+^-adaptive NK cells, derived from super donors—individuals with an innate genetic predisposition for highly reactive NK cell function—demonstrate superior alloreactivity toward HLA-mismatched AML [129]. These adaptive NK cells exhibit enhanced metabolic fitness, epigenetic reprogramming, and increased cytotoxicity, making them promising candidates for allogeneic NK cell-based immunotherapy.

Several commercial platforms are actively developing novel NK cell-priming methods to enhance the therapeutic efficacy of memory-like NK cells. INKmune™ (by INmune Bio) represents an innovative approach that does not require the ex vivo expansion or genetic modification of NK cells [124]. Instead, it primes endogenous NK cells in vivo, converting them into memory-like NK cells capable of sustained tumor surveillance and elimination across a broad spectrum of malignancies [130]. Recent studies have shown the therapeutic potential of WU-NK-101, an allogeneic cytokine-reprogrammed memory-like NK cell product, in relapsed/refractory AML [131]. The infusion of WU-NK-101 post-lymphodepletion showed notable immune modulation in the bone marrow microenvironment, linked to clinical outcomes. Furthermore, ongoing phase I clinical trials such as VOYAGE and APOLLO, initiated by Fate Therapeutics, are evaluating FATE-NK100, an investigational adaptive memory NK cell therapy derived from peripheral blood-expanded NK cells [132]. These studies are investigating its therapeutic efficacy in patients with AML and recurrent ovarian cancer, with preliminary results demonstrating its potential as a viable immunotherapeutic strategy. Table 1 provides an overview of companies actively developing memory-like NK cell-based therapies for cancer treatment.

Memory NK cells have emerged as a promising cellular platform for chimeric antigen receptor (CAR)-based immunotherapy, offering enhanced persistence, proliferative capacity, and cytotoxicity compared to their conventional NK cell counterparts. Preclinical data indicate that CAR-engineered CIML NK cells exhibit superior antitumor efficacy. Notably, CAR19 CIML NK cells co-expressing IL-15 have demonstrated improved tumor clearance and prolonged survival in lymphoma models [133]. Beyond CD19, other CAR constructs targeting tumor-associated antigens such as CD33, CD123, HER2, and ligands of NKG2D are currently under investigation for the treatment of AML and other malignancies. These approaches seek to leverage the inherent advantages of memory NK cells, like their rapid effector function and reduced risk of GvHD, while augmenting their antigen specificity, in vivo persistence, and resistance to immunosuppressive tumor microenvironments.

A phase I clinical trial, conducted by the Dana-Farber Cancer Institute (DFCI), is investigating the use of CIML NK cells in patients with platinum-resistant ovarian cancer [134]. The study administers CIML NK cells in combination with IL-2 to potentiate immune activation and enhance tumor cytotoxicity [135]. Another phase I trial by the DFCI explores the efficacy of CIML NK cells in patients with renal cell carcinoma and urothelial carcinoma, assessing IL-2’s role in augmenting NK cell-mediated tumor clearance [136]. Additionally, a phase I trial conducted by the Washington University School of Medicine is investigating CIML NK cell therapy in AML [137]. This study evaluates the persistence, proliferation, and functional efficacy of NK cells in inducing disease remission while monitoring their immunomodulatory effects. Beyond memory-like NK cells, allogeneic CAR-NK cell therapies are under evaluation for hematologic malignancies. A phase I/II trial at the MD Anderson Cancer Center assessed the therapeutic potential of HLA-mismatched anti-CD19 CAR-NK cells derived from cord blood in relapsed or refractory CD19-positive cancers, including non-Hodgkin’s lymphoma (NHL) and chronic lymphocytic leukemia (CLL) [138]. This study demonstrated a favorable safety profile, with no incidence of cytokine release syndrome, neurotoxicity, or graft-versus-host disease, without reaching the maximum tolerated dose. Of the 11 patients treated, 73% exhibited a positive response, with 7 achieving complete remission. The infused CAR-NK cells expanded in vivo and remained detectable at low levels for at least 12 months. Collectively, these trials underscore the therapeutic promise of NK cell-based immunotherapies, demonstrating high response rates, tumor regression, and durable remission across multiple malignancies. The safety profile remains favorable, with minimal toxicity observed. Table 2 summarizes ongoing clinical trials evaluating CIML NK cells and CAR-NK cell therapies across a spectrum of solid and hematologic malignancies, including ovarian cancer, renal cell carcinoma, and AML.

Current memory NK cell-based immunotherapies are predominantly limited to the use of CIML. However, antigen-specific NK cell memory, despite demonstrating promising therapeutic potential, remains largely confined to preclinical investigation. One of the key challenges in translating antigen-specific memory NK cells into clinical application is the technical difficulty associated with their large-scale ex vivo expansion, particularly with respect to achieving high fold-expansion rates and maintaining clinical-grade purity. CMV poses a serious threat to neonates and immunocompromised individuals, including cancer and hematopoietic stem cell transplant (HSCT) patients, due to its potential for severe reactivation and life-threatening complications [139]. Given the pivotal role of CMV-adaptive memory NK cells in antiviral immunity, their prophylactic or therapeutic use presents a compelling avenue for reducing CMV-related morbidity and mortality. Feeder cell systems, such as EBV-transformed lymphoblastoid cell lines and K562 erythroleukemia cells engineered to express membrane-bound IL-15 and 4-1BBL, have been shown to promote the expansion of NK cells with partial specificity for tumor-associated antigens [140]. These approaches have been incorporated into multiple clinical trials focused on hematologic malignancies. For instance, trial NCT00720785 evaluated NK cell therapy combined with bortezomib across various hematologic cancers, while NCT01904136 explored the use of haploidentical donor-derived NK cells in AML [141].

## 11. Conclusions and Future Directions

The discovery of NK cell memory represents a paradigm shift in immunology, challenging the classical view that immunological memory is exclusive to adaptive immune cells. In the case of CMV, a persistent viral infection in humans and mice, evolutionary pressures may have favored the expansion of NK cell subsets with receptors specifically tuned to recognize viral antigens. For example, NKG2C in humans and Ly49H in mice are expanded in individuals with chronic CMV infection, providing an enhanced potential evolutionary benefit by heightening viral control [142]. This selective expansion could be seen as an evolutionary adaptation, shaping NK cell receptor repertoires to improve responses to recurrent or persistent pathogens. Comparative evolutionary analyses reveal that NK cell receptor systems, including KIRs and CD94:NKG2A, co-evolve with their MHC class I ligands to regulate immune responses [143]. While CD94:NKG2A-HLA-E interactions are conserved, KIR interactions with classical MHC class I molecules (HLA-A, -B, -C) are highly polymorphic, driving NK cell diversity across human and primate populations [144].

High-dimensional profiling using mass cytometry (CyTOF) has revealed that HCMV infection induces broad phenotypic diversification within the NK cell compartment, associated with the selective expansion of adaptive NK cell subsets. However, this diversification appears to stem not from the de novo generation of receptor diversity, as in somatic recombination in T and B cells, but from the clonal expansion of pre-existing NK cell lineages bearing specific receptors. This suggests a model in which antigen exposure amplifies subsets with advantageous receptor profiles, effectively tuning the repertoire toward persistent pathogens without generating new receptor specificities. In this context, NK cell memory may be viewed as an evolutionarily selected trait that harnesses pre-existing receptor heterogeneity to confer enhanced protection against recurrent viral threats. The interplay between memory-driven clonal expansion and the evolutionary shaping of the NK receptor landscape represents a key area for further research, particularly in understanding how innate immune cells contribute to long-term immunological defense in an antigen-specific manner [145].

Besides viral infections, bacterial and parasitic pathogens, such as *Mycobacterium tuberculosis* and *Plasmodium* sp. (Malaria), have been linked to memory-like NK cell responses, suggesting a broader role for NK cells in pathogen-specific immunity. Additionally, NK cells have demonstrated functional relevance in autoimmune regulation and tissue repair, expanding their biological significance beyond traditional immune defense. CIML NK cells, generated by stimulation with IL-12, IL-15 and IL-18 exhibit enhanced and sustained IFN-γ production and cytotoxicity [146]. In contrast, virus-induced memory NK cells, such as those responding to CMV, rely on epigenetic modifications and antigen-driven expansion, leading to long-term persistence and enhanced ADCC [147]. While both NK cell types exhibit superior recall responses compared to conventional NK cells, CIML NK cells tend to be transient and dependent on continuous cytokine stimulation, whereas virus-induced memory NK cells persist long-term within the host. Recent advances in multi-omics technologies, including single-cell RNA sequencing (scRNA-seq) and single-cell ATAC sequencing (scATAC-seq), have revolutionized the characterization of memory NK cell subsets [148]. These approaches have revealed significant heterogeneity, distinct transcriptional profiles, and key epigenetic regulators of NK cell memory. Notably, the CD56^dim^/CD57^+^ NK cell subset has been identified as a marker of human memory-like NK cells, with RUNX3 emerging as a critical memory-stabilizing transcription factor [149].

Sex and aging have notable effects on NK cell biology, with emerging evidence suggesting that these factors may enhance NK memory functionality in females. Females typically exhibit stronger NK cell responses than males, with heightened spontaneous degranulation activity in intrahepatic NK cells during chronic hepatitis B, which correlates with elevated estradiol levels [150]. In aging populations, elderly women demonstrate more robust NK cell responses, including increased cytotoxicity and cytokine production, compared to their male counterparts. In males, aging is associated with the upregulation of genes involved in cytotoxicity and inflammation, while females show elevated expressions of genes linked to protein synthesis and cell junctions, indicating sex-specific regulatory mechanisms that may govern NK cell memory [151,152]. Single-cell transcriptomic analyses further reveal that aging exacerbates sex differences in NK cell gene expression [153].

Despite these advances, many fundamental questions remain unanswered. The precise molecular mechanisms underlying NK cell memory formation are still unclear. While virus-induced memory NK cells exhibit long-term persistence and antigen-specific expansion, CIML NK cells remain short-lived and lack antigen specificity. Understanding the mechanisms that regulate their survival, expansion, and persistence is essential for optimizing their therapeutic potential. Defining the molecular and transcriptional signatures that distinguish different memory NK cell subsets will enhance their application in immunotherapies and vaccine strategies. Additionally, the role of memory NK cells in tissue-resident immunity and their ability to provide long-term in situ protection against tumors or infections remains poorly understood. Investigating how memory NK cells interact with the tumor microenvironment, particularly in the presence of immunosuppressive factors such as TGF-β, could be pivotal in determining their efficacy in solid tumor therapies. Furthermore, metabolic adaptations that sustain NK cell memory require further exploration. Understanding how transitions among glycolysis, oxidative phosphorylation, and fatty acid oxidation impact NK cell longevity and function could pave the way for novel therapeutic interventions. Addressing these unanswered questions will be crucial for harnessing NK cell memory in the development of next-generation immunotherapies and vaccines.

The application of memory NK cells in immunotherapy is a particularly hopeful avenue, and several clinical trials are ongoing, with promising outcomes. Engineered CAR NK cells incorporating memory-like properties could enhance persistence and antitumor activity, potentially improving clinical outcomes in solid tumors and hematological malignancies. Induced pluripotent stem cell (iPSC)-derived memory-like NK cells represent a cutting-edge advancement in immunotherapy, merging iPSC technology with the potent functionality of memory NK cells. Their scalability and adaptability offer promising potential for both personalized and off-the-shelf NK cell therapies in diverse clinical applications. Moreover, combining memory NK cells with immune checkpoint inhibitors, metabolic modulators, or epigenetic drugs may optimize their efficacy in overcoming tumor immune evasion. Additionally, NK cell memory could play a crucial role in infectious disease control, particularly in vaccine development. Understanding how NK cells contribute to long-term immune protection may allow for the design of vaccines that stimulate durable NK cell responses, offering an alternative approach to traditional T- and B-cell-based immunity. Beyond infection and cancer, NK cell memory holds significant promise in regenerative medicine and transplantation. Tissue-resident memory NK cells have been implicated in wound healing and fibrosis regulation, suggesting their potential use in therapies aimed at promoting tissue repair. Additionally, NK cells could be leveraged to improve transplant outcomes by modulating graft rejection and tolerance mechanisms. Further research into how memory NK cells interact with tissue microenvironments will be essential for translating these findings into clinical applications.

## Figures and Tables

**Table 1:** Companies developing memory-like NK cell-based cell therapy products.

Sl. No.	Company Name	Platform/Product Name	Memory NK Cell Type	Cancer Targets	Development Stage	Description	Reference
1	Nkarta Therapeutics, San Francisco, CA, USA	NKX101	Engineered memory-like NK cells	AML, B cell malignancies	Phase I	CAR-NK cells with enhanced persistence and cytotoxicity	https://www.nkartatx.com/
2	Fate Therapeutics, San Diego, CA, USA	FT536	iPSC-derived memory NK cells	Solid tumors, NHL	Phase I	Multi-antigen-targeted CAR-NK with IL-15 boost	https://www.fatetherapeutics.com/
3	Affimed, Berlin, Germany	AFM13-NK	Cord blood-derived memory NK cells	Hodgkin’s lymphoma	Phase II (ANCHOR)	Innate cell engager (ICE^®^) combined with preactivated NK cells	https://www.affimed.com/
4	GT Biopharma, Brisbane, CA, USA	GTB-3550	TriKE-activated memory NK cells	AML, MDS	Phase I	Tri-specific NK engager (CD16/IL-15/CD33) to enhance NK memory response	https://www.gtbiopharma.com/
5	ImmunityBio, Culver City, CA, USA	haNK	IL-2/IL-15-primed memory NK cells	Solid tumors (pancreatic, NSCLC)	Phase II	Off-the-shelf NK cells with enhanced ADCC	https://immunitybio.com/
6	Artiva Biotherapeutics, San Diego, CA, USA	AB-101	Allogeneic memory-like NK cells	B cell malignancies, solid tumors	Preclinical/Phase I	IL-12/15/18-activated NK cells for improved tumor infiltration	https://www.artivabio.com/
7	Wugen, St. Louis, MO, USA	WU-NK-101	Cytokine-induced memory-like (CIML) phenotype	AML, glioblastoma	Phase I	Cytokine-induced memory-like (CIML) phenotype that supports enhanced antitumor activity, robust trafficking, superior proliferation capacity, and metabolic flexibility	https://wugen.com/
8	Glycostem Therapeutics, Oss, The Netherlands	oNKord^®^	Umbilical cord-derived memory NK cells	AML, ovarian cancer	Phase I/II	Proprietary expansion tech for high-persistence NK cells	https://www.glycostem.com/
9	Celularity, Florham Park, NJ, USA	CYNK-101	Placental-derived memory NK cells	Glioblastoma, AML	Phase I/II	Cryopreserved, off-the-shelf NK cells with cytokine augmentation	https://celularity.com/
10	Indapta Therapeutics, San Francisco, CA, USA	g-NK	Naturally occurring subset of natural killer cells known as g-NK	Rituximab in NHL, and in combination with daratumumab in multiple myeloma	Preclinical/Phase I	Universal, allogeneic NK cell therapy designed to improve treatment outcomes for cancer and autoimmune diseases, based on subset of NK cells known as g-NK cells	https://indapta.com/

Source: Official websites of each company are provided for reference. Web search conducted as of 1 May 2025.

**Table 2:** Global clinical trials using memory NK cells (2015–2024).

Sl No.	NCT Number	Study Title	Phase	Status	Memory NK Cell type	Conditions	Interventions	Sponsor	Study Type
1	NCT06321484	Intraperitoneal Cytokine-Induced Memory Like (CIML) NK Cells in Recurrent Ovarian Cancer	Phase I	Recruiting	Cytokine-Induced Memory-Like NK Cells	Platinum-Resistant Ovarian Cancer, Recurrent Ovary Cancer, Ovarian Cancer,	Interleukin 2	Dana-Farber Cancer Institute	Interventional
2	NCT06318871	Memory-like NK Cell Therapy in Patients with Renal Cell Carcinoma or Urothelial Carcinoma	Phase 1	Recruiting	Cytokine Induced Memory-Like Natural Killer (CIML NK) Cells	Renal Carcinoma, Renal Cell Carcinoma, Urothelial Carcinoma	Interleukin-2 (IL-2)	Dana-Farber Cancer Institute	Interventional
3	NCT06158828	Pilot Study of Memory-like NK (ML NK) Cells After TCRαβ T Cell Depleted Haploidentical Transplant in AML	PhaseI Phase II	Recruiting	Memory-Like NK (ML NK) Cells	AML, ChildhoodAML, Pediatric Acute Myeloid Leukemia	Rabbit Anti-Thymocyte Globulin; Drug: Busulfan; Drug: Fludarabine; 8 More	Washington University School of Medicine	Interventional
4	NCT06152809	CIML NK Cells With Venetoclax for AML	Phase I	Recruiting	Cytokine-Induced Memory-Like NK Cells, Interleukin-2	Acute Myeloid Leukemia, Recurrent Leukemia	Venetoclax	Dana-Farber Cancer Institute	Interventional
5	NCT06138587	Preemptive CIML NK Cell Therapy After Hematopoietic Stem Cell Transplantation	Phase I	Recruiting	Cytokine-Induced Memory-Like NK Cells, Interleukin-2	Acute Myeloid Leukemia, Myeloid Leukemia, 3 More	-----------	Dana-Farber Cancer Institute	Interventional
6	NCT05629546	Memory-Like NK Cells With Nivolumab and Relatlimab in Advanced or Metastatic Melanoma After Progression on Checkpoint Inhibitors	Phase I	Recruiting	Cytokine-Induced Memory-Like NK cells	Advanced Melanoma, Metastatic Melanoma	Relatilmab, Nivolumab	Washington University School of Medicine	Interventional
7	NCT05580601	Cytokine-Induced Memory-Like NK Cells (CIML-NK) for Relapsed & Refractory Acute Myeloid Leukemia (AML)	PhaseI Phase II	Recruiting	Cytokine-Induced Memory- Like NK cells	AML	CIML-NK Cells	Children’s Hospital Medical Center, Cincinnati	Interventional
8	NCT04893915	Cytokine-induced Memory-like NK Cells in Relapsed/Refractory AML and MDS	Phase II	Withdrawn	Cytokine-Induced Memory- Like NK cells	Relapsed AML, Refractory AML, Myelodysplastic Syndromes	Fludarabine, Cyclophosphamide	Washington University School of Medicine	Interventional
9	NCT04634435	Autologous Memory-like NK Cell Therapy With BHV-1100 and Low Dose IL-2 in Multiple Myeloma Patients	Phase I	Completed	Cytokine-Induced Memory-Like NK Cells	Multiple Myeloma	BHV-1100 Plus Cytokine-Induced Memory-Like (CIML) NK Cells plus IVIG and Low-Dose IL-2	Biohaven Pharmaceuticals, Inc.	Interventional
10	NCT04354025	Cytokine-induced Memory-like NK Cells in Combination with Chemotherapy in Pediatric Patents with Refractory or Relapsed AML	Phase II	Withdrawn	Cytokine-Induced Memory- Like NK cells	Refractory AML, Relapsed AML	Fludarabine, Ara-C	Washington University School of Medicine	Interventional
11	NCT04290546	CIML NK Cell in Head & Neck Cancer	Phase I	Completed	CIML NK	Squamous Cell Carcinoma of Head and Neck, Recurrent Head and Neck Squamous Cell Carcinoma	Interleukin-15 Superagonist (N-803), CIML NK Cell Infusion, Ipilimumab	Dana-Farber Cancer Institute	Interventional
12	NCT03068819	Cytokine Induced Memory-like NK Cell Adoptive Therapy for Relapsed AML After Allogeneic Hematopoietic Cell Transplant	Phase I Phase II	Recruiting	Cytokine-Induced Memory-Like (CIML) NK Cells	AML	CIML NK Cell Infusion, CD3+ T Cell Product Infusion	Washington University School of Medicine	Interventional
13	NCT02782546	Cytokine Induced Memory-like NK Cell Adoptive Therapy After Haploidentical Donor Hematopoietic Cell Transplantation	Phase II	Recruiting	Cytokine-Induced Memory-Like NK Cells	AML	Graft Cell Infusion, Tacrolimus, Mycophenolate Mofetil	Washington University School of Medicine	Interventional
14	NCT01898793	Cytokine-induced Memory-like NK Cells in Patients AML or Myelodysplastic Syndrome (MDS)	PhaseI Phase II	Terminated	Cytokine-Induced Memory-Like NK Cells	AML	Fludarabine, Cyclophosphamide, Leukapheresis	Washington University School of Medicine	Interventional

Source: https://clinicaltrials.gov/. Web search conducted as of 1 May 2025.

## Data Availability

No new data were created or analyzed in this study.
